# Vocal correlates of arousal in bottlenose dolphins (*Tursiops* spp.) in human care

**DOI:** 10.1371/journal.pone.0250913

**Published:** 2021-09-01

**Authors:** Rachel Probert, Anna Bastian, Simon H. Elwen, Bridget S. James, Tess Gridley

**Affiliations:** 1 Department of Agriculture, Engineering and Science, School of Life Sciences, University of KwaZulu-Natal, Durban, South Africa; 2 Sea Search Research and Conservation NPC, Cape Town, South Africa; 3 Department of Botany and Zoology, Faculty of Science, Stellenbosch University, Stellenbosch, South Africa; 4 Department of Statistical Sciences, Centre for Statistics in Ecology, Environment and Conservation, University of Cape Town, Cape Town, Western Cape, South Africa; Wildlife Conservation Society Canada, CANADA

## Abstract

Human-controlled regimes can entrain behavioural responses and may impact animal welfare. Therefore, understanding the influence of schedules on animal behaviour can be a valuable tool to improve welfare, however information on behaviour overnight and in the absence of husbandry staff remains rare. Bottlenose dolphins (*Tursiops* spp.) are highly social marine mammals and the most common cetacean found in captivity. They communicate using frequency modulated signature whistles, a whistle type that is individually distinctive and used as a contact call. We investigated the vocalisations of ten dolphins housed in three social groups at uShaka Sea World dolphinarium to determine how patterns in acoustic behaviour link to dolphinarium routines. Investigation focused on overnight behaviour, housing decisions, weekly patterns, and transitional periods between the presence and absence of husbandry staff. Recordings were made from 17h00 – 07h00 over 24 nights, spanning May to August 2018. Whistle (including signature whistle) presence and production rate decreased soon after husbandry staff left the facility, was low over night, and increased upon staff arrival. Results indicated elevated arousal states particularly associated with the morning feeding regime. Housing in the pool configuration that allowed observation of staff activities from all social groups was characterised by an increase in whistle presence and rates. Heightened arousal associated with staff presence was reflected in the structural characteristics of signature whistles, particularly maximum frequency, frequency range and number of whistle loops. We identified individual differences in both production rate and the structural modification of signature whistles under different contexts. Overall, these results revealed a link between scheduled activity and associated behavioural responses, which can be used as a baseline for future welfare monitoring where changes from normal behaviour may reflect shifts in welfare state.

## Introduction

Understanding and monitoring behaviour is a useful tool for welfare assessment [1] as abnormal behaviour may be indicative of poorer welfare [2]. However, the behavioural cues that animals give must be correctly recognised and interpreted by the observer. In captivity human caregivers may elicit behavioural responses from animals. Both positive and negative associations with human care givers have been observed where animals learn to associate humans with rewards and/or fear [3, 4]. In some livestock and poultry, human presence can induce fear as a result of stress and have a negative impact such as decreased growth rates [4, 5]. Alternatively, human presence and handling can improve welfare of captive animals, for example in weanling pigs [6] and beef calves [3]. Desired positive responses by animals toward human caregivers can be achieved by classical (involuntary) and operant (voluntary) conditioning through positive reinforcement [7]. Responses to human presence associated with feeding are often positive [8, 9] and accompanied by increased arousal [10]. The combination of feeding and handling has been shown to play an important role in the development of positive human-animal interactions [11]. Feeding time therefore seems to be a suitable candidate to study the behavioural response of a species to a positive interaction.

Animals housed in captive facilities tend to have structured daily schedules of events such as food provision or routine cleaning that are highly predictable through human-associated cues [12]. These may cause physiological and behavioural changes, reflecting anticipation and/or arousal. In general, arousal can be classified according to its level (high or low) and its valence (positive or negative) [13, 14]. Anticipatory behaviour preceding scheduled events are typically linked to elevated arousal states [9] and therefore can be used as an indicator of animal welfare through measuring the frequency of anticipatory-linked behaviours [15]. However, anticipation can be exhibited preceding events of both positive and negative valence [16] and may also present itself in different ways across individuals [17]. For example, in captivity, both increased and reduced activity have been demonstrated as anticipatory cues preceding regular events [18–21]. Therefore, information on baseline behaviour for each animal is necessary, from which behavioural adjustments may be indicative of a shift in welfare state [22].

Vocal expression of arousal is a common feature of communication in both humans and non-human animals [23–26] and can reflect responses to immediate experiences [8], or anticipation of rewards [19, 27, 28]. Vocalisations often carry prosodic cues about the arousal state of the sender [24] which makes the analysis of vocalisations a suitable tool to assess arousal states [25] and anticipation [29]. Dolphins live in groups with many social interactions, with vocalisations being an important method of communication within and between groups. The vocal repertoire of bottlenose dolphins consists of a range of pulsed and tonal sounds [30]. Tonal sounds include narrow-band, frequency modulated whistles to communicate during social interactions [31, 32]. Bottlenose dolphins use ‘signature whistles’ which are individually distinctive whistle types that encode identity information within their frequency modulation pattern and are the most frequently emitted whistle type produced by an individual [33]. Signature whistles are used to remain in contact [34–36] and address one another [37]. As might be expected for a cohesion call, individual production rates of signature whistles increase when motivation to maintain contact or return to the group is strong, i.e., during separation [36] and isolation events [38]. Although the whistle contour remains stable over time, shifts in whistle production rate, as well as frequency and duration characteristics, may reflect underlying arousal of individual dolphins [35, 38, 39].

Predictable human-controlled events such as feeding, public presentations, training sessions and medical examinations can evoke behavioural patterns in bottlenose dolphins [18] such as an increase in spy-hopping frequency and surface-looking before any human-animal interaction, including receiving of toys [40]. Vocal behaviour has been reported as highest when husbandry staff are present and during feeding/training sessions, and lowest at night when the numbers of caretakers are reduced [41] with peaks in activity overnight likely associated with bouts of social activity [42]. The morning arrival of husbandry staff has also been associated with anticipatory behaviour, as indicated by an increased vocalisation rate in captive false killer whales (*Pseudorca crassidens*) [29].

As vocal behaviour indicates underlying emotional states [8, 43, 44] and may be used as a non-invasive tool to monitor welfare for animals held in human care [45–47], the link between behavioural responses and daily events at a dolphinarium could provide insight into the animals’ life in human care. Therefore, the aim of this study was to investigate the overnight behaviour of bottlenose dolphins with a focus on patterns in detections and rates of whistling in response to early morning regimes including husbandry staff presence and feeding. We monitored the vocal response to scheduled events of the group as a whole, as well as at an individual level using signature whistles as animal personalities and responses to stimuli may vary [48, 49].

## Methods

All research undertaken was purely observational and research activities associated with hydrophone deployment (e.g., placing an object within the pool) were within the scope of normal activities conducted within the dolphinarium. The dolphins housed in this facility under human care is permitted by a South African Department of Environmental Affairs (DEA) permit (permit number confidential). No ethics clearance or permit was required for the acoustic monitoring of these animals as passive, non-invasive methodology was used and there was no direct contact with the animals.

**Fig 1:**
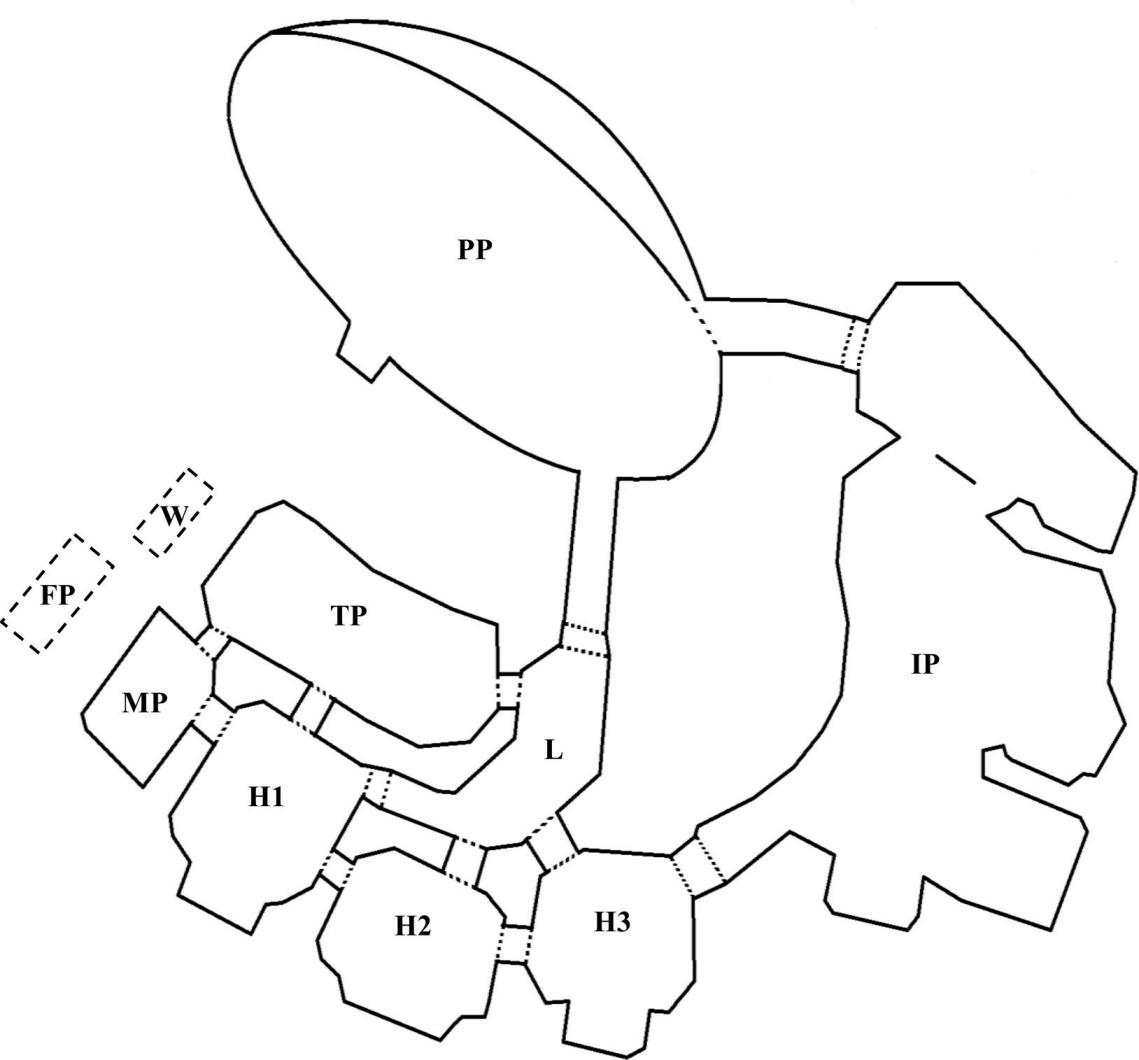
A schematic representation of the pools at uShaka Sea World (adapted from [51]). Presentation: PP- presentation pool. Back-of-house: TP–training pool, MP–medical pool, H1 –holding pool 1, H2 –holding pool 2, H3 –holding pool 3, IP–interaction pool, L–link channel. Workspace: W–work desk, FP–food preparation station.

This study took place at uShaka Sea World which was established in 2004 and is located in Durban, South Africa. The uShaka dolphinarium consists of a covered and open-air pool network with seven interconnected pools of varying size with a combined volume of 11 000 m^3^ (Fig 1). At the time of data collection in 2018, the facility housed ten bottlenose dolphins: three (two male and one female) common bottlenose dolphins (*T*. *truncatus*), one female Indo-Pacific bottlenose dolphin (*T*. *aduncus*) and six hybrids (four female and two male) of the two species [50]. The individuals were held in three social groups (see Table 1) separated by gates which allowed partial visual and full acoustic contact, but not free movement between social groups. All seven pools were utilised by the dolphins during the course of the study, with the number and configuration of pools within which social groups were housed varying throughout the days and overnight (Fig 1). As potential anticipatory- or arousal-eliciting activities, we noted morning activities at the dolphinarium which included arrival of husbandry staff (hereafter referred to as ‘staff’) and food preparation at 05h00 as well as feeding and vitamin administration at 06h00. Formal training occurred for 50–60 minutes per day per animal, outside of the study period and beginning after the morning feed. Between these training periods enrichment devices were available in all pools and trainers had numerous opportunities to informally engage with the animals. Trainers were mostly visible to the animals throughout the day as their workspace is situated poolside (closest to the medical and training pools, Fig 1). The last public presentation occurs from 15h00 to 15h30 and the last trainer leaves between 17h00 and 18h00, between which times all enrichment devices are removed from the pools. The range of dawn (06h04 – 06h25) and dusk (17h34 – 17h51) onset throughout the study period overlapped with morning feeding time and afternoon departure of staff.

**Table 1:** Genetic, social grouping and individual data for the dolphins at uShaka Sea World in 2018 (adapted from [50]).

Social group	Name	Species	Sex	Age in 2018 (years)	In captivity since	Parents	Signature whistle ID
Female group	Affrika	*Tt*	F	23	Captive born	P1 + *Tt*	F1
	Zulu	*Ta-Tt* Hybrid	F	19	Captive born	P1 + P2	F2
	Khanya	*Ta-Tt* F_2_	F	25	Captive born	M3 + deceased hybrid	F3
	Tombi	*Ta-Tt* Hybrid	F	25	Captive born	P1 + P2	F4
	Khethiwe	*Ta-Tt* Hybrid	F	9	Captive born	P1 + P2	F5
Male group	Ingelosi	*Ta-Tt* Hybrid	M	14	Captive born	P1 + P2	M1/M2
	Khwezi	*Ta-Tt* Hybrid	M	24	Captive born	P1 + P2	M1/M2
	Kelpie	*Tt*	M	34	Captive born	Wild *Tt* x deceased *Tt*	M3
Mixed group	Gambit	*Tt*	M	46	08/12/1976	Wild *Tt* x *Tt*	P1
	Frodo	*Ta*	F	44	26/06/1979	Wild *Ta* x *Ta*	P2

### Generating a signature whistle catalogue

The vocal behaviour of individuals was assessed through analysis of signature whistles. To determine the signature whistle for each dolphin, a catalogue was compiled using data collected during temporary isolation sessions in November 2016. Whistle contours are characterised by their time-frequency modulation patterns and in bottlenose dolphins, signature whistles can consist of a single contour or repeated contour (loop). A repeated contour, or multiloop whistle, is either connected where there are no breaks in the entire whistle contour or disconnected with a maximum inter-loop-interval of 0.25 seconds [39]. Acoustic data were collected using one to three dipping hydrophone(s) (HTI-96-MIN; High Tech Inc., United States; flat frequency response of 2 Hz– 30 kHz ± 1 dB) connected to a digital recorder (Tascam DR-680; TEAC America Inc., United States; sampling at 96 kHz and sample depth 24-bit stereo). Simultaneous vocal notes of observed behavioural data were recorded through a separate headset microphone. Time-frequency spectrograms of acoustic recordings (FFT = 1024, frequency range = 0–40 kHz, time series window = 10 seconds, Hann window, 50% overlap) were analysed in Adobe Audition CC (v 6.0; Adobe Inc., United States). Individual signature whistles were identified by isolating each dolphin from the others in a separate pool for 10–20 minutes while recording vocalisations. The signature whistle was defined as the most common whistle recorded during the temporary isolations and matched to that individual by comparing the relative amplitude of signals on the three hydrophones at different sites. Signature whistles were confirmed using the SIGID bout analysis method [33] whereby if stereotyped calls (minimum three out of four of the same type) occurring in a bout separated by 1–10 seconds were considered highly likely to be a signature whistle type and assigned to an individual.

### Investigating patterns of whistling behaviour

Acoustic data were collected over four periods in May, July and August 2018 using a compact acoustic recorder (Sound Trap 300 HF; Ocean Instruments, New Zealand; frequency response: 20 Hz– 150 kHz ± 3 dB, sensitivity: 183.3 dB re. 1 μPa) sampling the data at 576 kHz (16-bit mono). The acoustic recorder (hereafter “hydrophone”) was placed in the link channel (Fig 1), an area central to the pool network to which the dolphins did not have access but was within acoustic range of dolphins held in all pools. The hydrophone was attached to a 1 kg dive weight, suspended from a rope mid-water (at 1.5 m depth, channel depth 2.5 m) and held taut by attaching the rope to the facility roof. Ropes tied to the pool sides and roof were used to prevent movement which could produce unnecessary noise on the hydrophone. Acoustic recording commenced in the late afternoon after the final public presentation (between 15h30 and 17h00) and continued until 07h00 the following day. Recording was continuous, but files were restricted to standard 15-minute durations which constrained file sizes.

The first 30 minutes of each overnight deployment were discarded to obtain data unbiased by potential novelty effect as the dolphins may respond to the recorder’s presence in the water. Thereafter, one 15-minute file was selected to represent each hour from 17h00 to 07h00, using the recording which spanned the start of the hour. Each of the selected files were analysed in Adobe Audition CC by visually locating whistle contours in the spectrogram display (FFT = 1024, frequency range = 0–60 kHz, time series window = 10 seconds, Hann window, 50% overlap). Acoustic analysis and whistle classification was conducted through visual assessment by the lead author RP following well established methods [37, 52]. Each whistle was documented in a database and the signal to noise ratio (SNR) was visually assessed using the following criteria: SNR 1 –whistle is faint and barely visible, SNR 2 –whistle is clear and unambiguous, and SNR 3 –whistle is prominent [52]. Whistles of good quality (SNR 2 and 3) were either matched to the established signature whistle catalogue and categorised as a signature whistle from a specific individual or as ‘variable whistles’ containing all non-signature whistles from various individuals. The category ‘unclassified whistles’ was used for poor quality whistles (SNR 1) which did not match to the signature whistle catalogue (Table 2).

**Table 2:** Whistle categories included in the analyses and recording quality rating based on signal to noise assessment.

Whistle type	Description	SNR
Signature whistle	A whistle type unique to an individual that matches the signature whistle catalogue	2 & 3, but can include whistles with SNR 1 if we were confident that they are signatures
Signature whistle copying	Copying/matching of an individual’s signature whistle by another individual identified through overlapping of whistle contours	2 & 3
Variable whistle	Loud, unmasked whistles which do not match the signature whistle catalogue	2 & 3
Unclassified whistle	Masked or partial whistles that were unidentifiable and faint	1

In both the wild and captivity, bottlenose dolphins can copy the whistles of others. Such copies can be used in addressing or matching interactions, drawing attention from, or directing information to a particular individual [37, 53, 54]. In some cases, the whistle copy might not be an exact replication, but integrate features of the producer’s whistle type or voice [54], however in other cases copies may be indistinguishable from the original. Whistle copying is difficult to identify whistles with the same contour overlap in time, in which case we followed [55] in assigning the subsequent whistle as a copy and removing it from the signature whistle analyses but retaining these in the total whistle count. However, through the analysis process we identified stereotyped copying behaviour distinct from all other acoustic behaviour observed within the recordings. We noted that the square-shaped contour of one individual (P2) was emitted at various frequencies in prolonged series of copying interactions. For these stereotyped acoustic interactions, we could not confidently assign whistle production to P2, therefore we termed these whistle sequences ‘square copies’. These square copies, as well as all whistles 30 seconds before and after each event, were removed from the statistical analyses to prevent over-representation of individual P2.

To assess the production rate of whistles as a proxy of anticipation and/or arousal, we counted the number of whistles in each category (Table 2) per 15-minute recording. We investigated how whistle production rate (meaning all whistle types in Table 2 or only signature whistles) was affected by several covariates using a generalised additive mixed model (GAMM) approach using the package ‘gamm4’ version 0.2–6 [56] in RStudio version 4.0.3 [57]. This approach allows fitting of non-linear relationships between variables as well as the inclusion of both fixed and random effects. Whistle production was investigated in terms of both presence/absence and production rate (whistles per minute). A total of four models were built (two for all whistle types in Table 2, and two for signature whistles only) and for each, a combination of the following covariates were tested: ‘hour’ (hour of the day/night, continuous variable with non-linear smoothing term applied), ‘pool configuration’ (categorical variable with four levels indicating in which pools each social group was housed), ‘time of week’ (week vs weekend, categorical variable with two levels) and ‘presence of signature whistles’ (binary variable). Random effects for all four models included ‘sampling day’ to account for the variability of whistles production between sampling days. The selection of covariates was based on testing the assumption that patterns of activities and housing configuration in the dolphinarium may lead to different levels of anticipation and arousal which would reflect in vocal behaviour. Of most interest were the activities of the dolphinarium which occurred at scheduled times therefore ‘hour’ was of particular interest. Time of the week was of interest due to the increase of visitors to uShaka Sea World over the weekend. Codes were assigned to working days: 1 –‘week’, which consisted of Monday to Friday, and 2 –‘weekend’, which consisted of Saturday and Sunday. The covariate ‘pool configuration’ was of interest since visual contact between the different social groups or between groups and trainers (for example o husbandry staff arrival and food preparation) may affect levels of anticipation and arousal states. Pool configuration was coded into subcategories and defined according to which social group was housed in the presentation pool (PP; see Fig 1): Configuration 1 –all groups housed back-of-house with one group having access to the interaction pool (IP), and none having access to the PP; configuration 2 –the female group housed in the PP while the mixed and male groups housed back-of-house with one group having access to the IP; configuration 3 –the male group housed in the PP while the mixed and female groups housed back-of-house with one group having access to the IP; and configuration 4 –the mixed group housed in the PP while the male and female groups housed back-of-house with one group having access to the IP. The IP is directly linked to the PP, allowing the dolphins in each of these pools to be in visual contact through the gate. Configuration 1 is the configuration forming the intercept against which the other three configurations were compared against. All models were run using data between the hours spanning 17h00 – 07h00, with one extreme outlier (total whistle count = 276) removed from the total whistle and signature whistle production rate models.

We did not incorporate signature whistle ID as a covariate in the models of the full data series because the data were zero-inflated due to a lack of whistles emitted in most hours overnight. The models therefore investigate group, but not individual, calling behaviour. However, individual differences in vocal behaviour are of interest and relevant for animal welfare. We were interested in whether changes in individual calling behaviour could be explained by anticipation of staff arrival or increased arousal when staff were present and at feeding time. A fine scale look at the hours between 04h00 and 06h00 were used to investigate whether cues of anticipatory behaviour and/or arousal were reflected in vocal production rate. Therefore, a separate analysis was conducted to investigate individual differences between 04h00 and 06h00 (04h00 –one hour before staff arrival and food preparation; 05h00 –staff arrival and food preparation; 06h00 –feeding time). Data were not normally distributed and had unequal variances (Shapiro-Wilk test and Levene’s test, respectively) even after transformations (square-root, log and inverse transformations depending on the severity of the skew). Thus, Kruskal-Wallis tests were used with subsequent multiple Dunn *post hoc* tests following a significant result. We adjusted the alpha value using the Benjamini-Hochberg method to limit an increase in type I error rate, which is caused by alpha-inflation due to multiple pairwise comparisons.

By binning the time periods, we could investigate shifts in signature whistle production rate and structural characteristics relative to arousal responses to staff presence (05h00 – 07h00) compared to staff absence (20h00 – 04h00). For production rates, these were recalculated for each time period. Times 17h00 – 19h00 (staff transition period where staff were leaving and/or had just left) were omitted from this analysis due to the uncertainty of daily staff presence that occurred at these times. Data were not normally distributed and had unequal variances (Shapiro-Wilk test and Levene’s test, respectively) therefore two-tailed paired-sample Wilcoxon tests were used to investigate changes in production rate between the binned times. For signature whistle structural characteristics, we included individuals with sufficient data (minimum of five whistles in each binned time period) and selected five standard whistle parameters from signature whistles, namely: minimum and maximum frequency, frequency range (calculated from minimum and maximum frequency), duration and number of loops for each signature whistle. Measurements were taken from time-frequency spectrograms (FFT = 1024, frequency range = 0–40 kHz, time series window = 5 seconds, Hann window) of the fundamental frequency of a minimum of 33 and maximum of 392 signature whistles per animal in Raven Pro v1.6.1 [58]. Pairwise comparisons between the staff present and staff absent time bins were conducted for whistle parameters of the six individuals with sufficient data. Data were not normally distributed with unequal variances (Shapiro-Wilk test and Levene’s test, respectively), therefore two-tailed Wilcoxon tests were used to investigate changes in the acoustic parameters of signature whistles between binned times.

## Results

**Fig 2:**
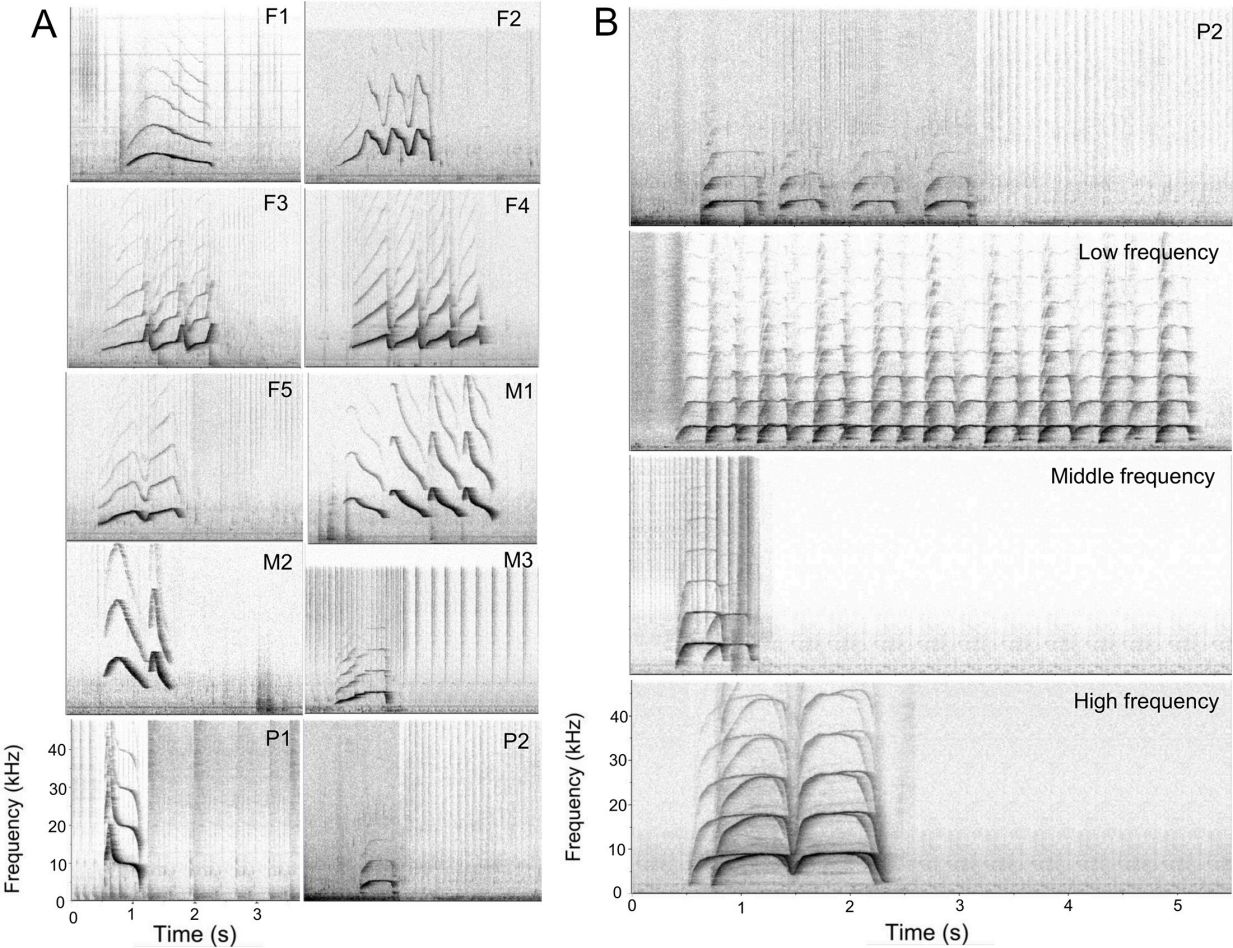
Whistle catalogues. (A) Signature whistle catalogue of all ten dolphins housed at uShaka Sea World in 2018. Individual identification codes are given in the top right corner of each spectrogram. Spectrograms of F1, M3, P1 and P2 show single loop whistles; F2, F3 and F5 show connected multiloop whistles; F4, M1 and M2 show disconnected multiloop whistles. (B) Signature whistle of dolphin P2 and examples of square whistle copying at different frequencies, with a similar contour shape to dolphin P2.

Nocturnal whistling behaviour was investigated from acoustic recordings made over 24 sampling nights (17 on weekdays and 7 on weekend days) which were conducted in four hydrophone deployment periods ranging from five to seven nights in duration. From this, 88 hours of acoustic data were analysed with a total of 2640 documented whistles, 1647 of which were signature whistles (62.4%), 168 variable whistles (6.3%), 569 unclassified whistles (21.6%), 3 signature whistle copying (0.1%) and 253 square whistle copying (9.6%) (Table 2). A total of ten signature whistles were documented for the group of ten dolphins, of which eight were assigned to individuals (see Table 1, Fig 2A). The two remaining signature whistles could not be confidently differentiated between individuals M1 and M2, which are hybrid siblings that engage in a significant amount of whistle copying. All square whistle copying sequences were removed from the analyses (Fig 2B).

Square whistle copying behaviour was uncommon and occurred sporadically with no particular temporal trend (S1 Fig). Furthermore, these square copying events occurred predominantly during the night when overall signature whistle production was at a minimum. A closer inspection of these square whistle copying events indicated that the vocal behaviour was more involved than a simple owner-copy interaction. In the 30 seconds before and after these whistle copying events that occurred throughout the data set, whistles were produced most frequently by three of the animals (F5, M1 and M2; S2 Fig). After removing all whistles 30 seconds before and after each square whistle event, 2281 whistles, 1561 of which were signature whistles, were included in the analyses.

**Fig 3:**
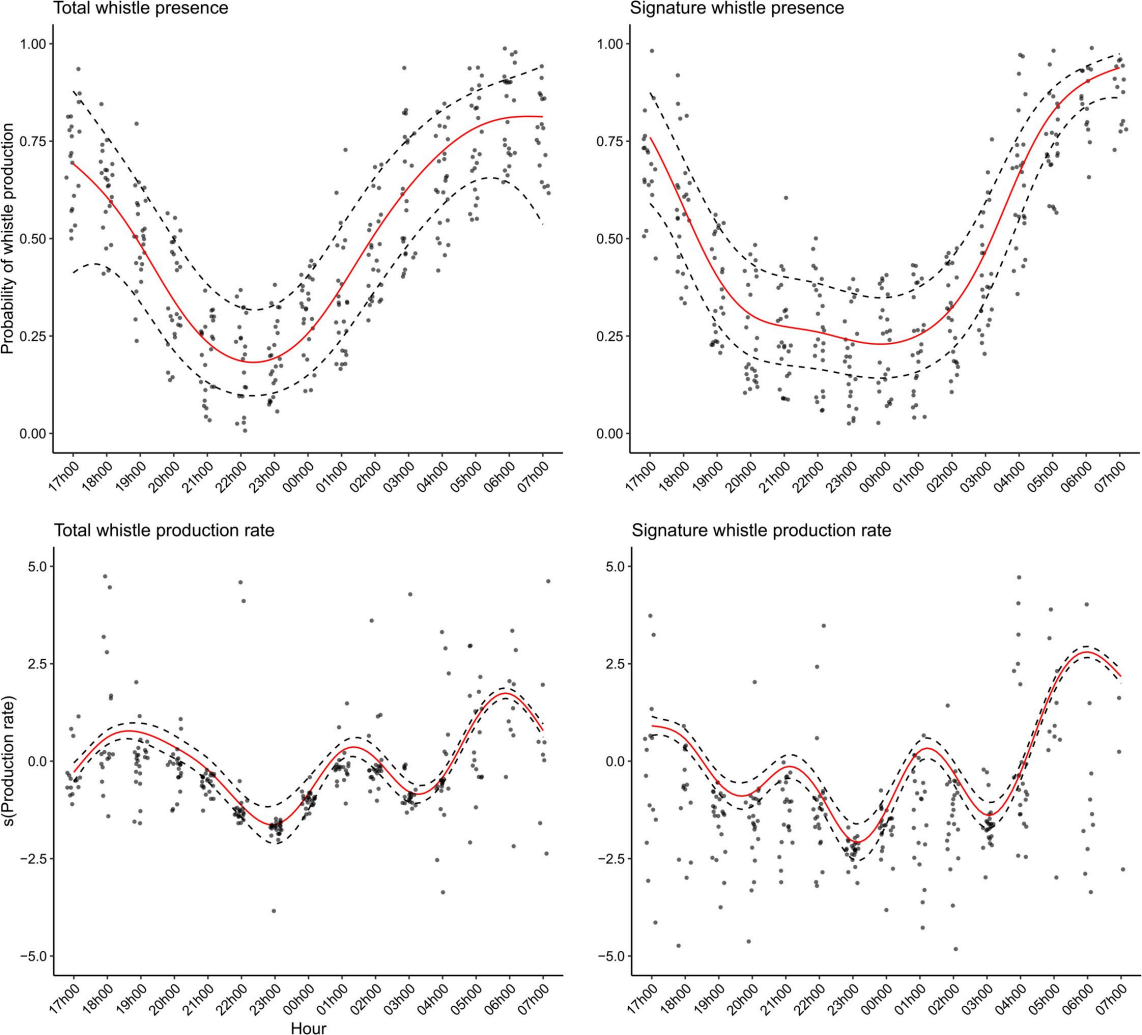
Presence and production rate of whistles and signature whistles over time. This graph shows the GAMM summary of smoothing term ‘hour’ for all four models used in this study. The y-axis represents the log scale of whistle production rate occurring at each time bin.

**Fig 4:**
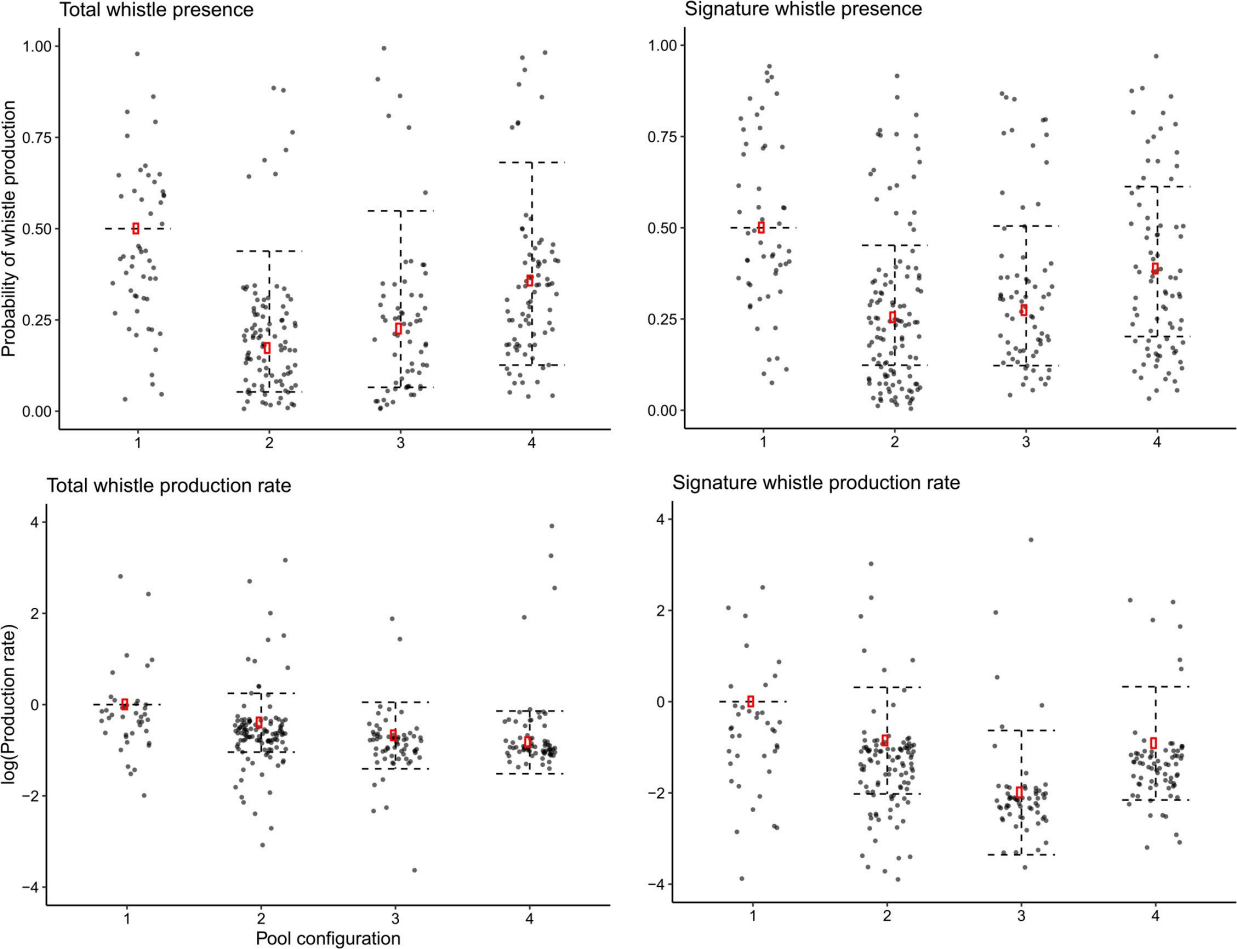
Presence and production rate of whistles and signature whistles compared between pool configurations. This graph shows the GAMM summary of the covariate ‘pool configuration’ for all four models. The y-axis represents the log scale of whistle production rate occurring at each time bin.

The GAMMs show a temporal trend in presence of total whistles and signature whistles with two clear peaks, one in the late afternoon and one in the morning (Fig 3; Table 3 –Models 1 and 3; variable ‘hour’; *p* < 0.001). The morning peak corresponds to staff presence, particularly to the time of arrival of staff and preparation of food in the morning (05h00), which is prior to the onset of dawn (06h04 – 06h25) and remains high during feeding (06h00). The late afternoon/evening peak occurs during the time that husbandry staff were leaving the facility (17h00 – 18h00) and overlapping dusk (17h34 – 17h51). Patterns in whistle production (total whistles and signature whistles) are more variable, however both show a clear morning peak at 06h00 (Fig 3; Table 3 –Models 2 and 4; variable ‘Hour’; *p* < 0.001). Model output showed that pool configuration is an important factor in all four models, where presence and production rates were greatest when all social groups were housed back-of-house (Fig 4; Table 3 –Models 1 to 4; variable ‘Pool configuration’; *p* < 0.05). However, the results were somewhat mixed. Compared to the intercept of configuration 1 (all groups housed back-of-house), total whistle and signature whistle presence decreased when the female group was housed in the presentation pool (configuration 2). On the other hand, when compared to configuration 1, whistle production rate was lowest when the mixed group was housed outside (configuration 4) and signature whistle production rate was lowest when the male group was housed outside (configuration 3). The likelihood of whistle or signature whistle occurrence per hourly time bin did not differ between weekdays and the weekend, however signature whistle production rate was greater over the weekend (Table 3 –Model 4, variable ‘Time of week’). Although not significant, the inclusion of the ‘time of the week’ variable increased model performance (R-squared from 0.178 to 0.233). Total whistle production is highly dependent on the presence of signature whistles (Table 3 –Model 2; variable ‘Signature whistle presence’; p < 0.001). Although not significant in Model 1, this variable significantly increased overall model performance (R-squared from 0.302 to 0.649). The low to average adjusted R-squared values in three of the four models (Table 3 –adjusted R-squared range = 0.233–0.521) indicate that other factors also influence whistle and signature whistle production that cannot be explained by the covariates in these models.

**Table 3:** GAMM results for four models (only *p-*values < 0.05 are presented).

Response variable	N	Explanatory variables				Adjusted R-squared
		Hour	Pool configuration	Time of week	Signature whistle presence	
**Model 1: Whistle presence**	347	< 0.001	0.042 (Intercept)	NA	-	0.649
			0.020 (config 2)			
**Model 2: Whistle production rate**	346	< 0.001	0.044 (Intercept)	NA	< 0.001	0.521
			0.025 (config 4)			
**Model 3: Signature whistle presence**	347	< 0.001	0.034 (Intercept)	NA	NA	0.279
			0.017 (config 2)			
**Model 4: Signature whistle production rate**	346	< 0.001	0.001 (Intercept)	-	NA	0.233
			0.008 (config 3)			

**Fig 5:**
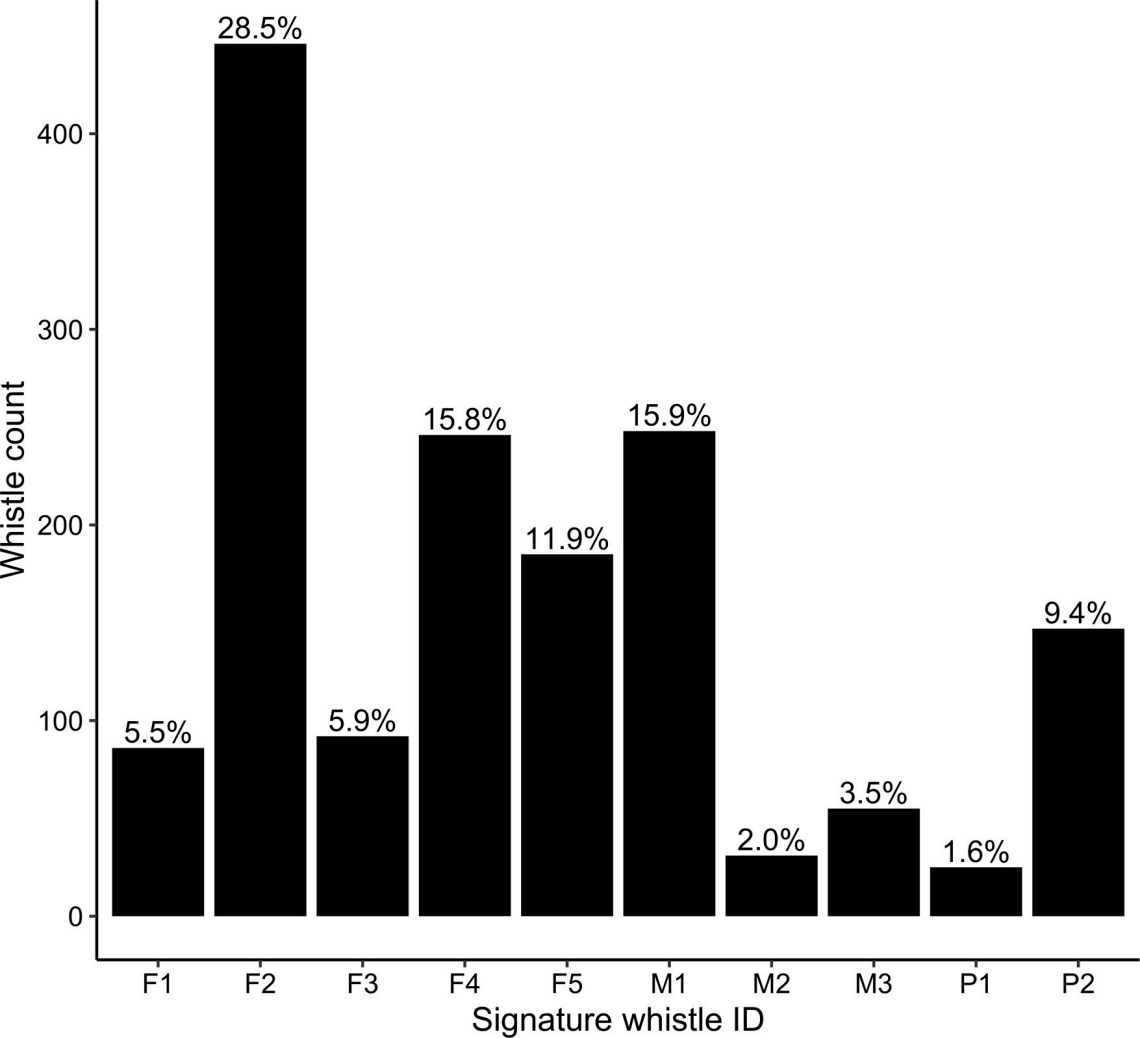
Total count of signature whistles produced by individual dolphins throughout the study period.

**Fig 6:**
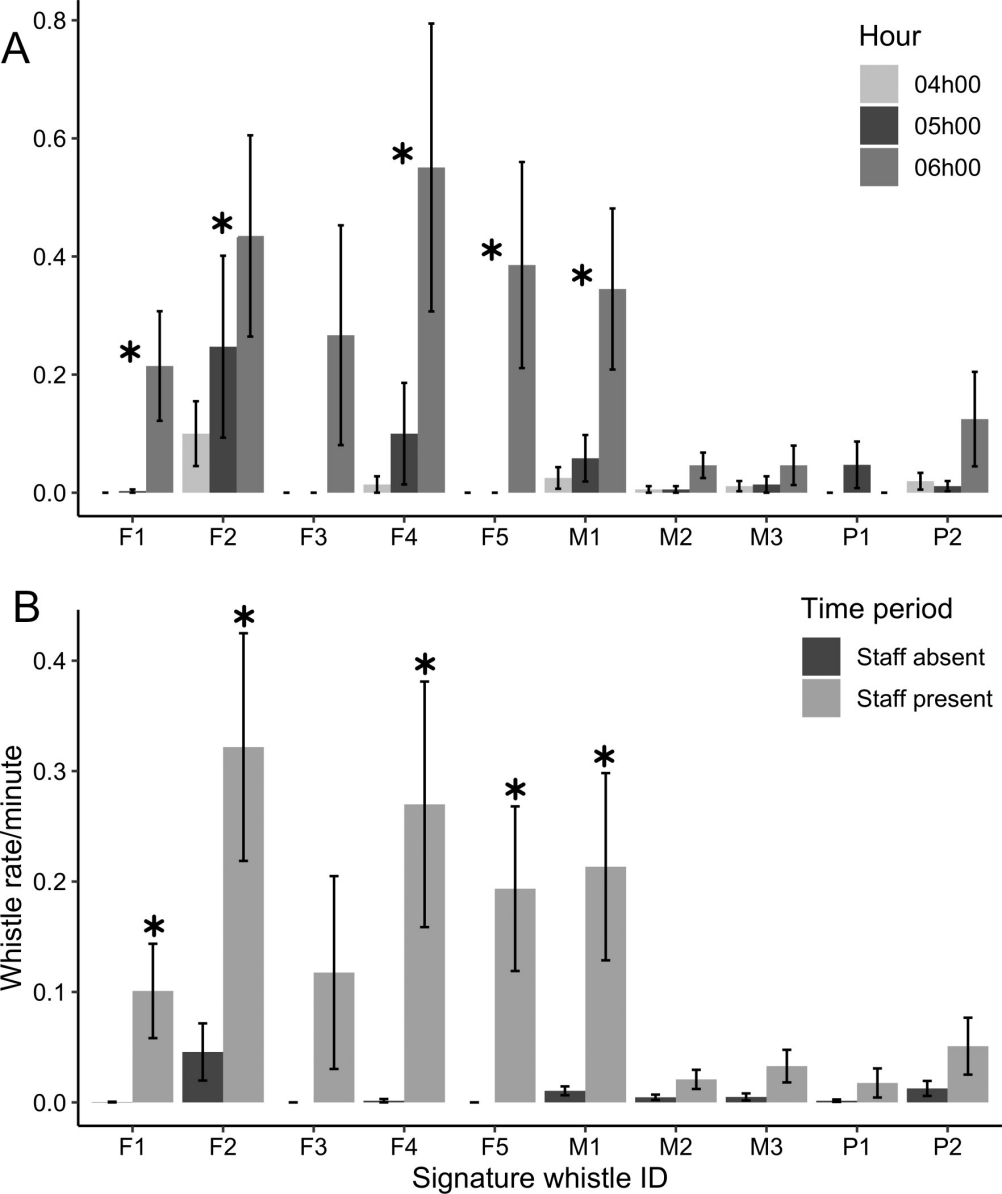
Individual vocal responses to feeding and staff presence. (A) Signature whistle production rate of each individual compared across three hours (mean (SD)). Significance indicated by asterisks is consistent among individuals, with no significant shift between 04h00 and 05h00 only. (B) Whistle production rate of all individuals with staff absent and present (mean (SD)). Significance between time periods indicated by asterisks.

There was a large difference in signature whistle production between individuals. Of all the signature whistles included in the analysis ~ 60% were assigned to three individuals (two female and one male, Fig 5). The three least vocal animals, contributing a combined ~ 7% to the total signature whistle production, were all males (Fig 5). A fine scale look at signature whistle production rate between 04h00 and 06h00 demonstrated that for all animals apart from P1, signature whistle production was highest at 06h00 (feeding) compared to 04h00 (staff absent) and 05h00 (staff present), a difference which was significant for five of the 10 dolphins ((Fig 6A; p < 0.05, n = 866). This behavioural shift was most obvious for dolphins F1, F3, F5 and M2 whereby signature whistle production rates were zero or near-zero between 04h00 and 05h00 and increased steeply at 06h00. Overall whistling rates were low for individual P1 (Fig 5), and it was unusual that he produced signature whistles occasionally at 05h00 and not at all during feeding at 06h00.

**Fig 7:**
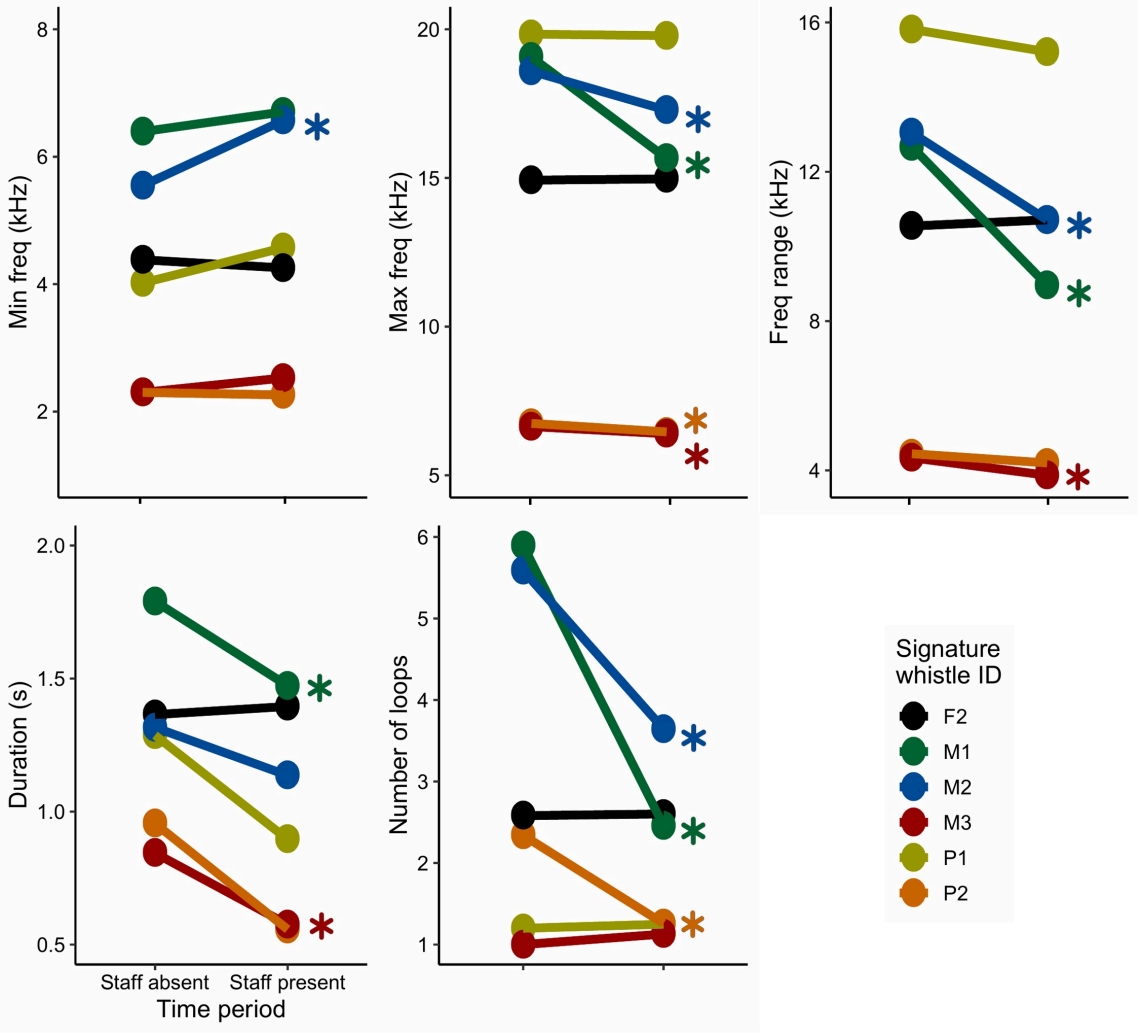
Shifts in signature whistle parameters in response to staff presence. Asterisks indicate significance between the two time periods.

By binning the time, we could take a closer look at individual behaviour in relation to staff absence (20h00 – 04h00) and staff presence (05h00 – 07h00). Signature whistle production from all of the dolphins was higher when staff were at the facility and pairwise comparisons indicated that this shift was significant for five of the animals; the same five animals that exhibited a strong response to feeding (Fig 6A and 6B; p < 0.05, n = 1413). In the absence of staff, bouts of whistling occurred sporadically between sample days, driven by half of the animals. Sufficient data were available for six dolphins to investigate changes in signature whistle time-frequency characteristics between periods of staff absence and presence (Fig 7). Two individuals including the most vocal dolphin in the analysis (F2) exhibited high degrees of structural stability with no significant shifts in any whistle characteristics when staff were present compared to absent. However, in four animals there was a reduction in in maximum frequency and in turn, a reduction in frequency range when staff were present (Fig 7). For two animals there was a significant reduction in whistle duration when staff were present and for three animals, this was reflected in a significant reduction in the number of loops.

## Discussion

This study demonstrated shifts in the vocal behaviour of captive bottlenose dolphins associated with heightened arousal in response to staff presence and feeding at uShaka. The strongest result obtained was the fundamental shift between non-whistling and whistling behaviour observed from overnight to the morning. The prominent shift in presence and production rate of whistles and signature whistles was paired with structural changes in the characteristics of signature whistles in some individuals, including a general decrease in whistle duration and maximum frequency. Although the vocal patterns coincided with staff departure and arrival at the facility, the timing was not independent from onset of dusk and dawn, therefore our results are not independent of (or mutually exclusive from) changes in light availability. Previously, behavioural changes in response to light availability were documented [59], however, due to positive reinforcement, staff arrival and feeding are strong drivers of behavioural response in captive cetaceans [29, 60, 61], and may have a greater effect on behaviour than changes in light availability due to the food reward. We did not demonstrate anticipatory vocal behaviour preceding staff arrival and food preparation as whistle production rates remained relatively low in the two hours preceding the morning feed. On testing whether husbandry decisions impact whistle presence and production, we found that total whistle and signature whistle production rates were greatest during the morning period when all social groups were housed together back-of-house and in visual contact with staff. Although signature whistles are primarily used as a contact call during individual or group separation [34, 36] we did not detect a vocal response associated with housing configurations where groups were more separated. As higher whistle production occurred when all animals were house inside back-of-house together and in view of staff activity, this rather suggests a group response to visual cues associated with food provision. Additionally, whistle production rate was higher over the weekend compared to weekdays however this result was not significant. While the number of visitors were greater on weekends, the daily schedule was the same every day which may explain the weak relationship.

The overall whistle production overnight was very low reflective of previous studies demonstrating that resting accounts for up to 87% of nocturnal behaviour of captive bottlenose dolphins [62]. However, bouts of whistling behaviour occurred during the night on some of the recording nights, as previously documented in captive bottlenose dolphins [63]. Resting is associated with various behaviours, including synchronous swimming, a behaviour observed in both the wild and captivity [62] as well as at the uShaka dolphinarium [51]. Bottlenose dolphins increase social interactions before some rest periods, possibly to promote synchronous swimming [62], and may be reflected in the bouts of whistling behaviour we observed overnight. The social dynamics are different among the three social groups housed at uShaka and although housed separately, they are always in acoustic (and mostly visual) contact. This may allow inter-group social bonds to be formed, which are prevalent in some captive mammals [64, 65]. The square whistle copying interactions we identified are likely a learned behaviour, which may serve as an underlying social function for the dolphins at uShaka, much like the shared group whistles documented in closely allied dolphins [66, 67]. However, without associated behavioural observation overnight, we cannot assign a cause or context to the infrequent bouts of nocturnal whistling and square whistle copying interactions.

Food-anticipatory activity describes increased arousal before feeding events on strict daily schedules [10] and has been frequently observed in captivity [41, 61]. We found that whistle and signature whistle production rates slightly increased (but not significantly) in the morning during staff arrival and food preparation, significantly intensified during feeding (in five of the dolphins) and decreased one hour after feeding (Fig 6A). Such increased vocalisation rates during feeding and when husbandry staff are present has been documented for cetaceans at other captive facilities [29, 61]. As the strongest behavioural response was directly associated with feeding rather than during the phases of staff arrival and food preparation, our results indicate that the arousal is directly associated with feeding rather than an anticipatory vocal behaviour. However, as our data were point sampled and rounded to the closest hour, expression of anticipatory behaviour immediately preceding feeding events remains possible.

Vocal responses characterised by similar vocal features may indicate both positive and negative arousal states [17] as previously indicated in wild bottlenose dolphins [39, 68, 69]. Unlike indicators of arousal, recent literature comparing indicators of valence suggest that this may be more species-specific [70]. Previously, predictability of events in captive facilities has resulted in conflicting conclusions regarding animal welfare [see 71], and both positive [72] and negative [12] behavioural responses were demonstrated by animals in response to feeding. Excitement is an example of a positive arousal state [14, 73], and is less commonly studied than negative arousal. The morning increase in vocal behaviour at feeding time that we observed may suggest positive arousal associated with a food reward whereby the animals are exhibiting excitement to this particular event. However, if the feeding signal is inaccurately given, this could lead to arousal associated with frustration [12]. Although the morning feed at uShaka occurs at the same time each day, the increased vocal production cannot be directly attributed to a positive or negative arousal state, particularly due to the individual variation in behavioural response at this time.

Signature whistles are considered structurally stable over time [34], however subtle shifts in signature whistle structural characteristics have been demonstrated in the wild and in captivity [35, 38, 74]. During the temporary capture of wild *T*. *truncatus*, [39] found that the animals increased signature whistle production and the number of whistle loops at the beginning of capture, which slowly decreased over the course of the brief event. Additionally, calves produced whistles with greater maximum frequencies when briefly captured with their mothers. These characteristics were indicative of a stress response in temporarily captured dolphins. At uShaka, most individuals responded to staff presence by increasing whistle production and some by reducing the number of loops and maximum frequency. Signature whistle production rate may therefore be indicative of overall arousal in bottlenose dolphins however valency may be indicated in underlying structural characteristics. Furthermore, individual differences in vocal behaviour were observed among all animals studied which may relate to personality differences [48, 49], experience (two animals were wild-born and eight captive-born) or more general differences related to sex [75], age and species [38, 39]. Although limitations in our sample size prevent any detailed investigation of most of these factors, we did note a possible effect of sex in the study. Of the four males housed at uShaka, three were notably quiet, accounting for only 7% of the overall signature whistle count data. It was also the males which exhibited the greatest shift in whistle frequency and duration characteristics related to periods of staff presence and absence. Therefore, our study indicates that male and female dolphins exhibit differential responses to arousal which warrants further exploration at other captive facilities and in the wild.

The behaviour observed in one facility may not match the behaviour observed in another as groups of bottlenose dolphins may develop their own behavioural patterns through transmission of socially learned behaviour [see 76]. Likewise, our results, as well as results from [38], demonstrate that vocal responses across individuals is varied, which emphasises the limitations of a unified approach to monitoring welfare and the importance to explore individual states [22]. Understanding individual behavioural baselines in whistle production and characteristics provides a useful index from which abnormal changes could be indicative of a shift in welfare state. Traditionally welfare assessments in the past were heavily focussed on monitoring behaviour indicative of poorer welfare as good welfare was thought to result from lack of suffering [77]. Although our study does not indicate whether increased arousal reflects positive or negative emotional states associated with food provisioning, it provides a useful index for the individuals housed at uShaka.

## Supporting information

S1 Fig Total number of square whistle copying events during each time bin throughout the study period. (TIF)

S2 Fig Total whistle counts, as well as P2 as the initiator of the copying events [55]. (TIF)
