# Supplementary figures and images for: Vocal correlates of arousal in bottlenose dolphins (Tursiops spp.) in human care
Source: PLoS One. 2021 Sep 1;16(9):e0250913. doi: 10.1371/journal.pone.0250913 (PMC8409691; doi:10.1371/journal.pone.0250913)

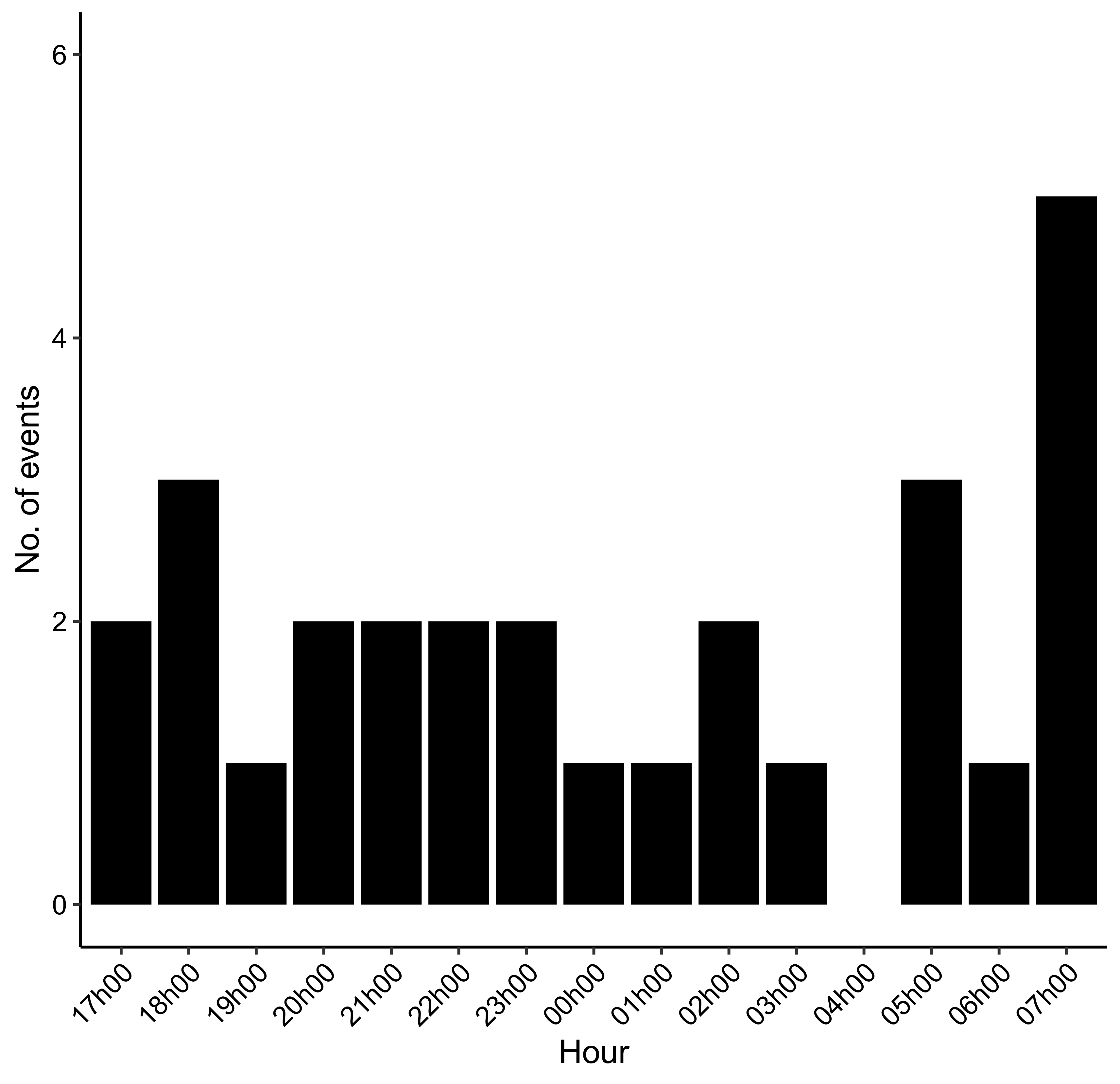

Supplement: S1 Fig — (TIF) [file pone.0250913.s001.tif]

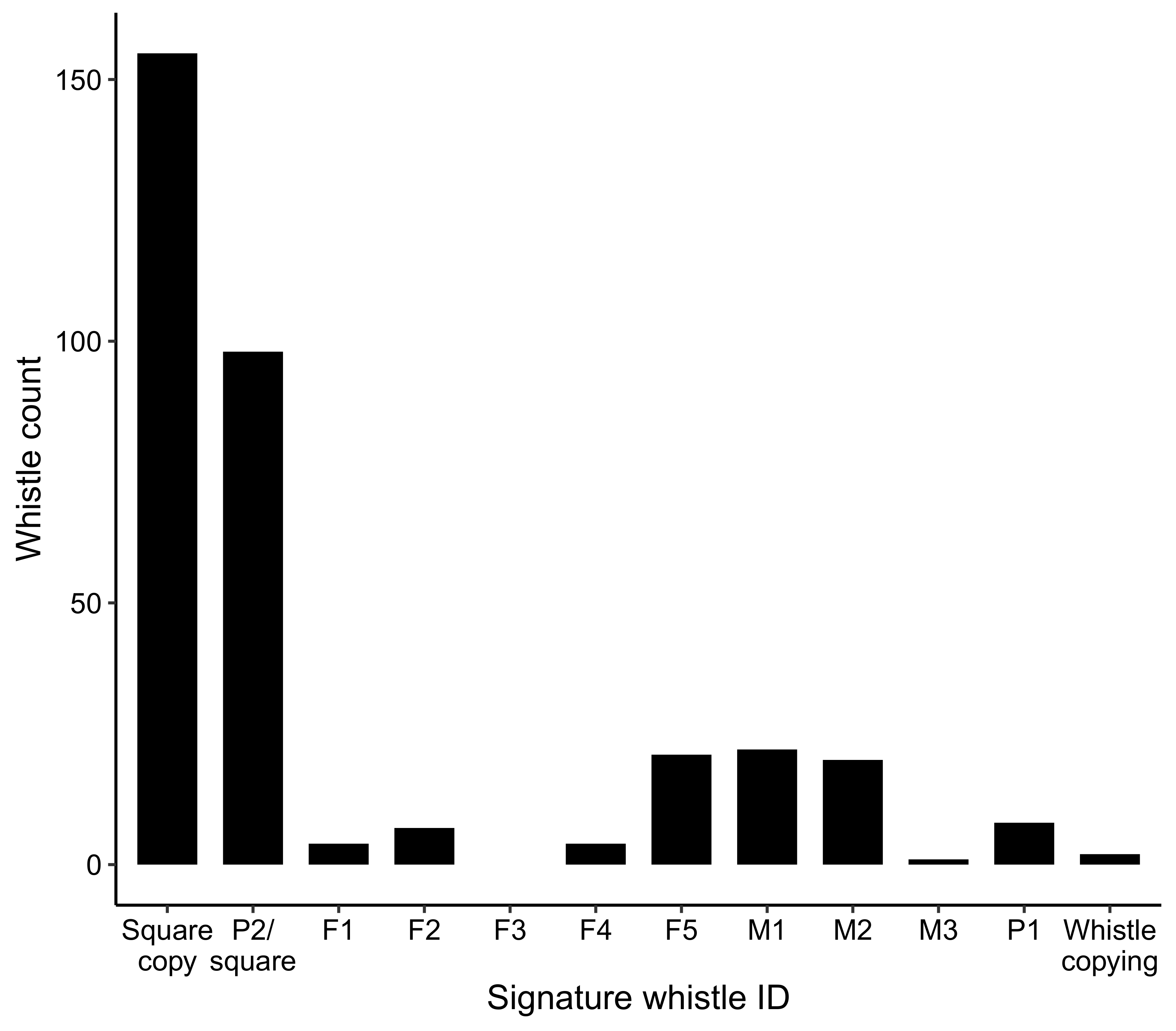

Supplement: S2 Fig — (TIF) [file pone.0250913.s002.tif]
